# Intersectional forces of urban inequality and the global HIV pandemic: a retrospective analysis

**DOI:** 10.1136/bmjgh-2023-014750

**Published:** 2025-04-09

**Authors:** Ingrid T Katz, Dana Renee Thomson, Sindhu Ravishankar, Kennedy Otwombe, Erlyn Rachelle Macarayan, Carissa Novak, Alison R Schulte, Sidney Atwood, Liana Rosenkrantz Woskie, Zoe Siegel, Bruce D Agins, Janan Dietrich, Blair T Johnson, Erva-Jean Stevens, Lisa M Butler, Matthew Kavanagh

**Affiliations:** 1 Mass General Brigham, Harvard Medical School, Cambridge, Massachusetts, USA; 2 University of Twente Faculty of Geo-Information Science and Earth Observation, Enschede, Overijssel, The Netherlands; 3 Center for International Earth Science Information Network, Columbia Climate School, New York, New York, USA; 4 International Association of Providers of AIDS Care, Washington, District of Columbia, USA; 5 Fast-Track Cities Institute, Washington, District of Columbia, USA; 6 Perinatal HIV Research Unit, Faculty of Health Sciences, University of the Witwatersrand, Johannesburg, South Africa; 7 School of Public Health, Faculty of Health Sciences, University of the Witwatersrand, Johannesburg, South Africa; 8 Brown University School of Public Health, Providence, Rhode Island, USA; 9 Harvard Global Health Institute, Cambridge, Massachusetts, USA; 10 Global Health and Population, Harvard T H Chan School of Public Health, Boston, Massachusetts, USA; 11 International Division, JSI Research and Training Institute Inc, Boston, Massachusetts, USA; 12 Brigham and Women’s Hospital, Boston, Massachusetts, USA; 13 Tufts University Department of Community Health, Medford, Massachusetts, USA; 14 Brown University, Providence, Rhode Island, USA; 15 Institute for Global Health Sciences, University of California San Francisco, San Francisco, California, USA; 16 Perinatal HIV Research Unit, University of the Witwatersrand Johannesburg Faculty of Health Sciences, Johannesburg, Gauteng, South Africa; 17 Health Systems Research Unit, South African Medical Research Council, Cape Town, South Africa; 18 University of Connecticut Institute for Collaboration on Health Intervention and Policy, Storrs, Connecticut, USA; 19 Joint United Nations Programme on HIV/AIDS, Geneve, Switzerland; 20 Public Health Sciences, Queen’s University School of Medicine, Kingston, Ontario, Canada; 21 Global Health, Georgetown University, Washington DC, District of Columbia, USA

**Keywords:** Public Health, Global Health, HIV

## Abstract

To determine how the intersection of increased urban growth and poverty has impacted HIV incidence and prevalence, given growing HIV inequalities globally. Retrospective analysis using combined data from five publicly available, population-level datasets to determine city- and within-urban countrywide estimates of 95-95-95 treatment targets, prevalence and incidence rates from 2015 to 2019. For city-level estimates, we analysed combined data from: Fast-Track City (FTC), SINAN from Brazil and UNAIDS Naomi-Spectrum. Countrywide estimates of HIV prevalence in the urban slum versus non-slum since 2012 were compiled from Population-Based HIV Impact Assessment (PHIA) surveys in 12 countries and Demographic Health Surveys (DHS) in 28 countries. HIV prevalence is generally higher among the urban slum, compared to their non-slum counterparts, thus resulting in national HIV estimates masking nuances in HIV inequalities between the urban slum and non-slum. Specifically, national and city-level HIV estimates mask inequalities within and between cities, with secondary cities often having higher HIV prevalence and incidence rates than capital cities and large urban areas. The urban divide between slum and non-slum populations is a contributor to HIV inequality, often with poorer outcomes in smaller cities than their larger counterparts. Interventions tailored to cities, and particularly those considering local nuances in subpopulations (eg, different genders, ages, roles), are necessary to reduce HIV inequality. Focused HIV programming accounting for structural drivers of inequalities between urban slum and non-slum populations such as inequalities in wealth, education, employment and housing are crucial to closing gaps driving HIV inequalities globally.

**Summary box**

- There is a global rise in growth among urban slum populations in the world, and cities remain the epicentre of the global HIV pandemic.
- Subnational variation in HIV burden has been well documented with some cities faring better than others.
- There is a demonstrated link between structural inequalities such as inequalities in education, housing and income, and inequalities in health outcomes.
- We found that HIV prevalence is higher among the urban slum populations compared with urban non-slum counterparts, which ultimately results in national estimates of HIV masking nuances in HIV inequalities between the two populations
- We found that large secondary cities (1–5 million population) often have equal or greater HIV incidence and prevalence compared with major cities.
- These data provide a framework for focused HIV programming crucial to closing gaps driving HIV inequalities globally.

## Introduction

 Approximately 56% of the world’s population currently lives in urban areas, and that proportion is expected to increase to 68% by 2050.^1^ Ninety per cent of projected urban growth will be in African and Asian cities alone, with a disproportionate increase among the poorest.^1^ The SARS-CoV-2 pandemic has compounded this trajectory by increasing the number of people who are newly poor within a short timeframe.^2 3^ Cities also remain the centre of the HIV pandemic globally, with a single city accounting for up to 30% of a country’s HIV burden in some cases.^4^ This pattern has emerged despite that the many advantages cities have in offering cost-effective HIV service infrastructure and resources.^5^ Understanding the intersectional forces of rising urban inequality with the global HIV pandemic is crucial to reaching the 95-95-95 targets and ending the global HIV epidemic by 2030, as set forth by the latest UNAIDS Global AIDS Strategy 2021–2026.^6^

The conceptual link between HIV and intersecting facets of inequality in urban contexts is longstanding; however, it was largely studied in high-income countries and before the era of treatment-as-prevention. Early studies in the 1980s and 1990s identified a link between poverty and HIV in urban concentrated epidemics in high-income countries,^7 8^ and subsequent studies in the early 2000s across generalised epidemics in African countries showed the opposite—that HIV infection is higher among wealthier individuals.^9^,^11^ Recent findings have indicated that the association between wealth and HIV has weakened over time and that urbanicity is a confounding factor.^12^ Urban issues and challenges are particularly important to consider, given the rapid expansion of cities and continued evolution of urban population dynamics.^13^ For example, in the last three decades, the number of megacities has more than tripled. The determinants of socioeconomic inequalities in the global HIV pandemic, particularly in low- and middle-income countries (LMICs), are poorly understood; however, previous studies in Sub-Saharan Africa have found that inequality is often a more important risk factor for HIV prevalence than actual wealth at the aggregate level.^14^ The intersectional forces of economic inequality on other marginalised identities and social positions globally can have a compounding effect.^15^ In 2021, key populations such as men who have sex with men, people who inject drugs, transgender people, and sex workers and their clients accounted for 70% of HIV infections globally.^16^

Geographic factors, such as urban residence, also factor prominently. Prior research using the data from the Demographic Health Survey (DHS) and AIDS Indicator Surveys to measure inequalities in HIV prevalence in 24 countries in Sub-Saharan Africa suggested that HIV is more prevalent among relatively wealthier countries and individuals within the region.^17^ However, within urban areas in countries such as Uganda, Kenya, Zimbabwe and Swaziland, HIV was more prevalent among the poor.

This study sets out to explore the intersectional HIV inequalities in contemporary cities based on a common absolute definition of poverty and deprivation, recognising that cities are highly heterogeneous and disease and social dynamics in giant global megacities are likely quite different from those in small regional hubs. We used data from publicly available, population-level datasets to examine HIV incidence and prevalence variability at the intersection of urbanity and poverty (thus referred to as urban slum and non-slum) and to answer the following research questions: What is the variation in HIV incidence and prevalence across large and smaller urban areas globally? What is the variation in HIV incidence and prevalence within urban areas between urban slum and non-slum populations?

## Methods

We performed a retrospective analysis using data from city-level and individual-level public datasets to determine city- and within-urban estimates of prevalence and incidence rates (from 2015 to 2019). For city-level estimates, we analysed combined data for 222 cities across UNAIDS-defined regions where we had data for >10 cities. Resulting regions included: Eastern and Southern Africa (98 cities); West and Central Africa (83 cities); Latin America and the Caribbean (18 cities); and Western and Central Europe and North America (23 cities). We did a search of public domain city databases and identified three sources: Fast-Track City (FTC) database with incidence and prevalence directly reported by city authorities (primarily surveillance data), SINAN with directly reported prevalence from Brazilian city authorities and UNAIDS Naomi-Spectrum subnational modelled estimates in Africa. Prevalence and incidence data were extracted for all cities that reported it, and all cities in the regions that had >10 cities were included in the analysis. The Naomi-Spectrum estimates are produced by administrative units, so we used administrative unit estimates where they were geographically aligned with city boundaries. UNAIDS Global AIDS Monitoring and the AHEAD database from the USA were also considered but not included because cities either had a more recent direct estimate from FTC or SINAN or a more recent modelled estimate in Naomi-Spectrum. Countrywide estimates of HIV prevalence in urban slum versus non-slum populations (since 2012) were compiled from Population-Based HIV Impact Assessment (PHIA) surveys in 12 countries and DHS in 28 countries. PHIA and DHS follow similar robust multistage sampling designs, use of standardised questionnaires and protocols, biometric testing for HIV, and standard data management and cleaning protocols.^18 19^

### Statistical methods

City prevalence and incidence indicators were spatially joined to city boundaries from the Functional Urban Areas dataset by the European Commission and mapped.^20^ The Naomi-Spectrum estimates modelled by administrative units were matched to Functional Urban Area boundaries in a Geographic Information System using visual inspection. The Functional Urban Areas dataset included population estimates for 2015, which were used to classify cities by population size based on Dijkstra *et al*^21^ and whether it was a capital city, as follows: capital/extra-large (>5 million); large (1 million–5 million); medium (250 000–1 million); and small (<250 000). We refer to capital cities and cities with more than 5 million people as ‘major cities,’ and all other cities as ‘secondary cities.’ Prevalence and incidence rates were compared across city types within regions (where we had data for at least 50 cities) using the t-test statistic, and p values less than 0.1 were interpreted as indicating a potential difference.

To understand within-city disparities, HIV prevalence estimates were calculated from PHIA and DHS survey data sets by urban ‘slum’ and urban non-‘slum’ households. Incidence data were not available in a majority of these surveys and thus are not reported in this analysis.

‘Slum’ households are defined by UN-Habitat as lacking improved water, improved sanitation, durable floor or sufficient space.^22^ Although ‘slum’ households are not necessarily located in areas with informal settlement, this asset-based definition is a strong proxy of the urban poorest and populations living in the most deprived areas of cities. The ‘slum’ household definition is an absolute measure of poverty that is measured with the same assets consistently across countries and over time.

Mean prevalence estimates were calculated by ‘slum’/non-‘slum’ household type applying sampling weights specific to individuals interviewed about HIV in each survey and plotted with 95% CIs by country and region accounting for clustering. Statistical analysis was conducted using SAS Enterprise Guide,^23^ STATA 17^24^ and Python.^25^ Spatial data management, analysis and mapping were performed in ArcGIS 10.8.^26^

## Results

**Figure 1:**
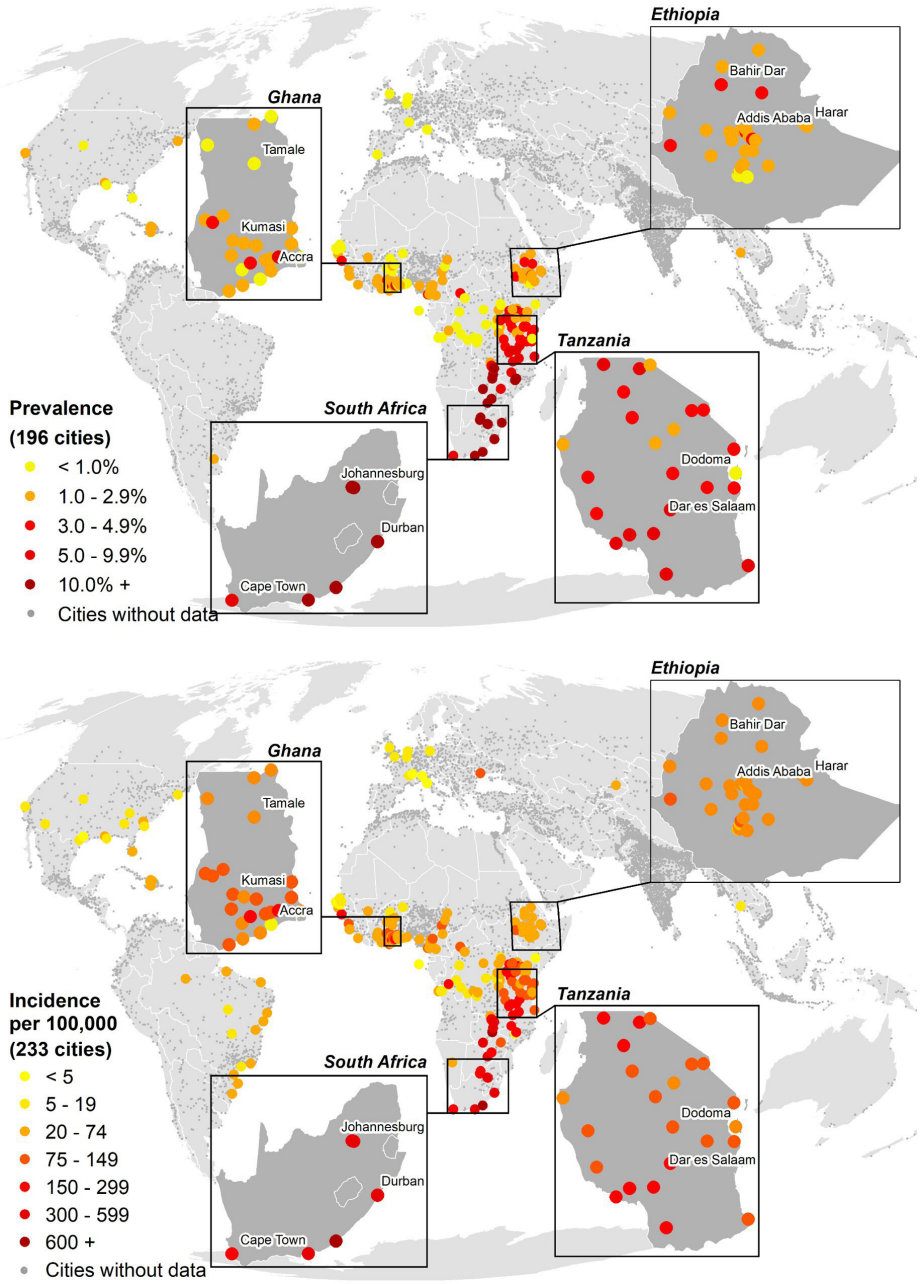
HIV prevalence (top) and incidence (bottom) since 2015, by city. Sources: Naomi-Spectrum model estimates (81% of prevalence and 91% of incidence data), Fast-Track Cities direct reports (13% of prevalence and 9% of incidence data) and SINAN direct reports (6% of prevalence data).

The maps in figure 1 underscore the importance of disaggregating HIV indicators beyond national to the city scale where there is large variability in prevalence and incidence rates. In Tanzania, for example, the Naomi-Spectrum model estimated a prevalence of 0.8% and incidence of 25 cases per 100 000 people in Zanzibar City (population 700 000), 9.0% prevalence and 242/100 000 incidence in Makambako (population 70,000), and 3.9% prevalence and 116/100 000 incidence in Dar es Salaam (population 5.6 million). Similar disparities were observed across cities in Ethiopia, Ghana and other countries. We found that capital cities and other major cities (ie, 5 million or more population) did not always experience the greatest HIV burden per capita; in many cases, large secondary cities (ie, 1–5 million population) had similar or higher HIV prevalence and incidence rates, though these patterns differ by region.

In Eastern and Southern Africa (ESA), the mean prevalence of HIV in major cities was 6.5% on average compared with 11.8% in large cities (p<0.05), but no differences in mean prevalence were detected between major cities and secondary cities with fewer than 1 million residents (medium, 4.9%, p >=0.1; small, 4.6%, p>=0.1) (table 1). Mean HIV incidence followed a similar pattern in this region with 247 cases per 100 000 in major cities and a higher, but not statistically different rate, in large cities (314/100 000, p>=0.1). Unlike the prevalence pattern, however, incidence rates were lower in secondary cities of less than 1 million people compared with major cities (medium, 127/100,000, p<0.1; small, 131/100 000, p<0.1) (table 1).

**Table 1:** City prevalence and incidence by city population and region

City type	Prevalence				Incidence				Interpretation
	N	Mean	SD	t-test	N	Mean	SD	t-test	
Africa: Eastern and Southern	97	5.34	4.54		97	156	213		
Capital/XL (5M+)	14	6.54	4.36	Ref.	14	247	451	Ref.	A similar proportion of people are living with HIV (prevalence) in small- and medium-sized as capital/XL cities, and large cities have the largest proportion of people living with HIV (greater than capital/XL cities). Additionally, more people are testing positive (incidence) in large, XL, and capital cities than in small- or medium-sized cities.
Large (1 M–5M)	5	11.76	4.74	5.22*	5	314	135	67	
Medium (250 k–1M)	26	4.92	4.71	−1.62	26	127	147	−120✝	
Small (<250 k)	52	4.62	4.04	−1.92	52	131	126	−116✝	
Africa: West and Central	81	1.45	1.09		83	50	49		
Capital/XL (5M+)	20	1.57	1.08	Ref.	20	51	66	Ref.	Prevalence and incidence rates are not substantially different in capital/XL cities than smaller non-capital cities, though a larger sample size of cities may be needed to detect differences.
Large (1 M–5M)	10	1.44	0.88	−0.13	10	52	32	<1	
Medium (250 k–1M)	20	1.03	0.83	−0.54	22	31	24	−21	
Small (<250 k)	31	1.65	1.27	0.08	31	64	51	12	
Latin America and Caribbean	5	1.50	0.38		17	34	14		
Capital/XL (5M+)	2	1.60	0.56	N/A	3	25	2	N/A	Insufficient sample size to evaluate differences across city types.
Large (1 M–5M)	0	--	--		7	37	19		
Medium (250 k–1M)	2	1.60	0.28		5	35	10		
Small (<250 k)	1	1.10	--		2	33	19		
West and Central Europe, North America	12	0.68	0.44		22	14	10		
Capital/XL (5M+)	4	0.68	0.32	N/A	5	18	13	N/A	Insufficient sample size to evaluate differences across city types.
Large (1 M–5M)	4	0.44	0.22		7	12	6		
Medium (250 k–1M)	4	0.93	0.64		8	15	10		
Small (<250 k)	0	--	--		2	10	10		

Key: *p<0.05, ✝p<0.1.

Note: The regions of ‘Asia and Pacific’ and ‘Eastern Europe and Central Asia’ are not reported because we had prevalence and/or incidence data for only two cities in each region. The following countries had a large number of cities in the analysis which might influence results: USA (13), Tanzania (23), Kenya (13), Ghana (24), Ethiopia (28), DR Congo (21), Cameroon (12) and Brazil (13).

In West and Central Africa (WCA), no statistical differences were detected in HIV prevalence or incidence in major versus secondary cities of any size (table 1), though several secondary cities had similar incidence and prevalence as major cities. Mean prevalence was 1.57% in major cities, compared with 1.44% (p>=0.1) in large cities, 1.03% (p>=0.1) in medium cities and 1.65% (p>=0.1) in small cities. However, comparatively lower levels of HIV infection in this region make differences more difficult to detect. We did not perform statistical comparisons among cities in Latin America and the Caribbean or in Western and Central Europe and North America because we had data on relatively few cities, and levels of HIV infection are relatively lower in these regions; however, mean prevalence and incidence across city types do not vary widely (table 1). While it is known that key population epidemics, for example, among sex workers, people who inject drugs, and men who have sex with men, account for large segments of the HIV epidemic in many of these cities,^16^ analysis of subpopulation epidemics is outside the scope of this analysis.

**Figure 2:**
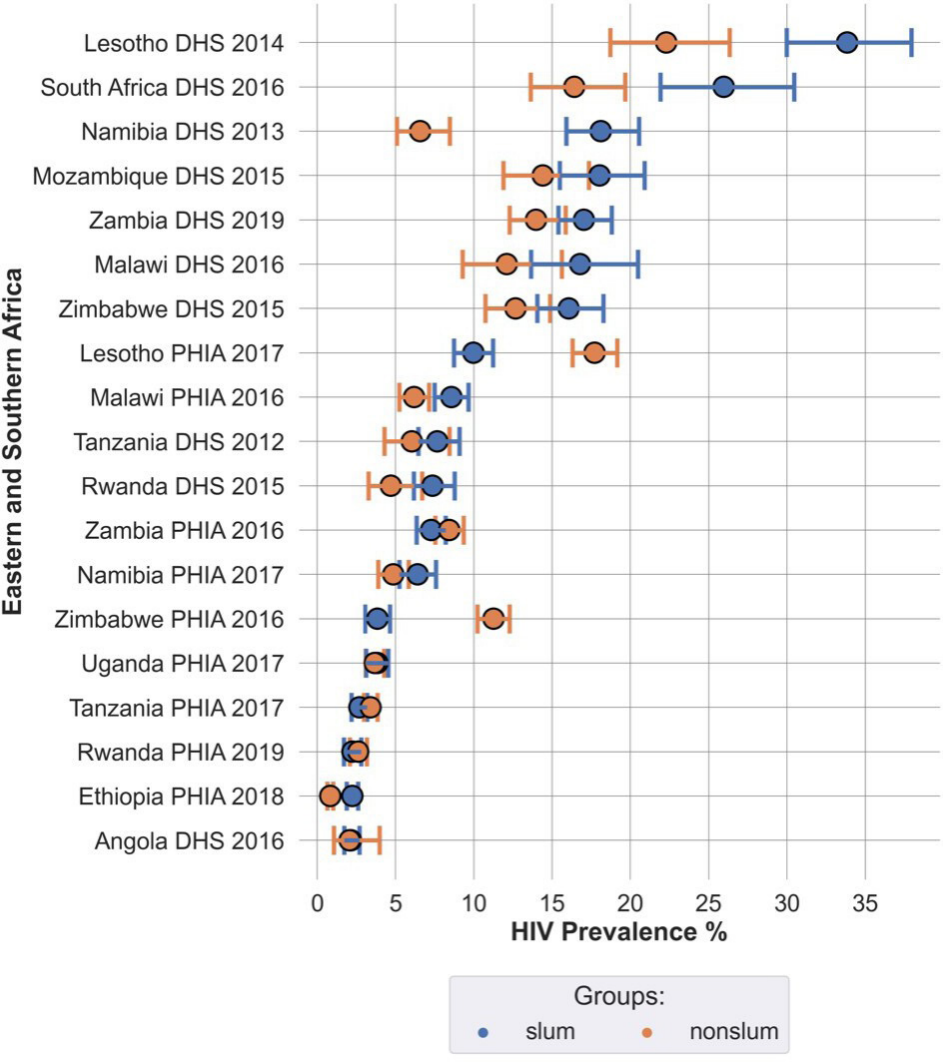
Prevalence of HIV in Eastern and Southern African Countries by setting (urban ‘slum’ and non-‘slum’). DHS, Demographic Health Surveys; PHIA, Population-Based HIV Impact Assessment.

**Figure 3:**
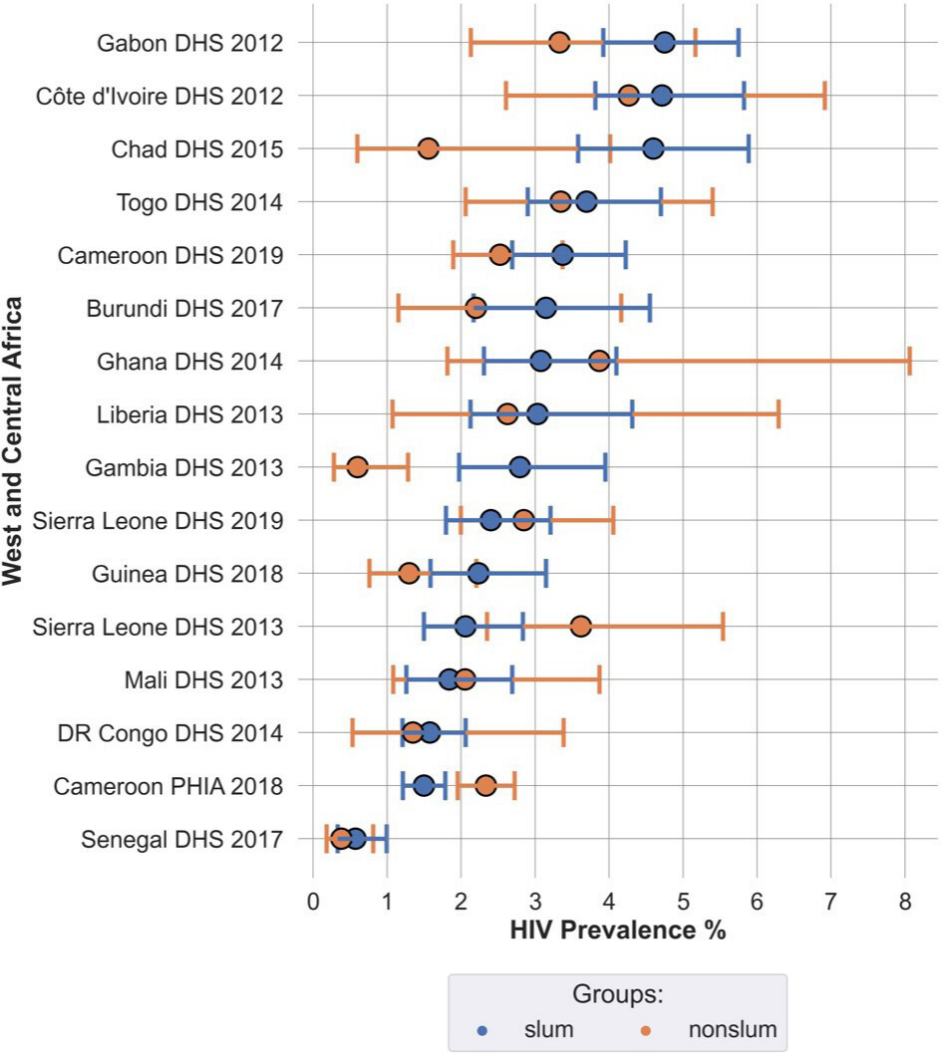
Prevalence of HIV in West and Central African Countries by setting (urban ‘slum’ and non-‘slum’). DHS, Demographic Health Surveys; PHIA, Population-Based HIV Impact Assessment.

Figures2^4^ show HIV prevalence across four regions of the world where DHS or PHIA data are available since 2012. Countries are ordered by prevalence among urban ‘slum’ populations from highest to lowest. Within urban areas, we found that HIV prevalence is generally higher among ‘slum’ residents especially in Eastern and Southern Africa (Ethiopia, Lesotho, Malawi, Namibia, Rwanda, South Africa, Zambia, Zimbabwe) as well as Latin America (Dominican Republic and Haiti). Although there were several surveys in which non-‘slum’ dwellers had a higher prevalence of HIV than ‘slum’ dwellers (Zambia, Zimbabwe, Tanzania, Lesotho, Cameroon), these were all PHIA surveys, and all countries had another survey which measured higher prevalence among ‘slum’ dwellers at a different point in time (figure 3, onlinesupplemental tables S1  S2). We hypothesise in the Discussion about discrepancies between DHS and PHIA surveys and the directionality of urban inequalities, though what is clear is that HIV had a disproportionate burden on the most deprived and vulnerable ‘slum’ households.

**Figure 4:**
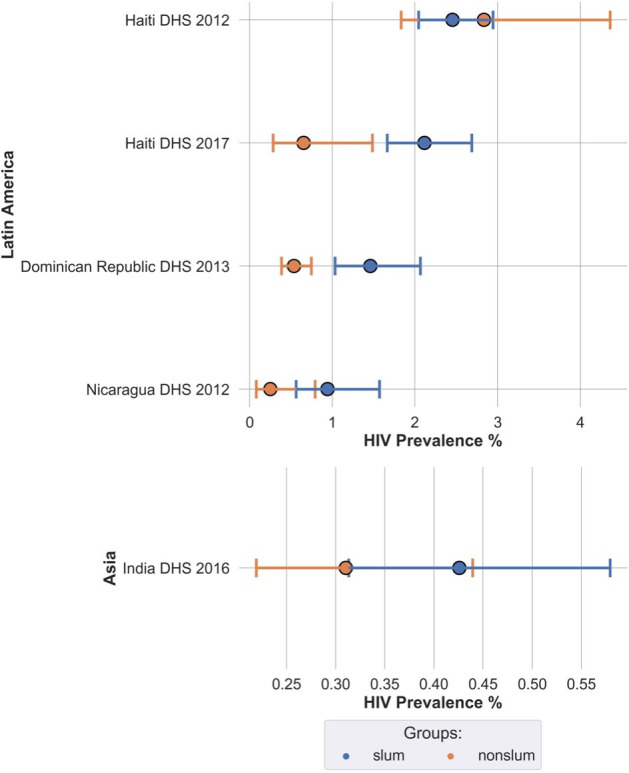
Prevalence of HIV in Latin America and Asia by setting (urban ‘slum’ and non-‘slum’). DHS, Demographic Health Surveys; PHIA, Population-Based HIV Impact Assessment.

In ESA, most countries except for Namibia and Malawi had higher HIV prevalence among ‘slum’ compared with non-‘slum’ populations. Meanwhile, in Burundi, Ethiopia, Malawi and Uganda, ‘slum’ and non-‘slum’ populations had similar HIV prevalence rates. Burundi, Ethiopia, Uganda and Angola had smaller differences in HIV prevalence between ‘slum’ and non-‘slum’ groups. Overall, narrower differences in HIV prevalence were observed across economic groups in East African countries, whereas disparities were greater in Southern African countries.

In WCA, there were few statistical differences in HIV prevalence between ‘slum’ and non-‘slum’ populations; however, the overall trend was higher HIV prevalence in ‘slums’ compared with non-‘slums’ in eight out of 13 countries (62%): Chad, Gambia, Gabon, Guinea, Togo, Liberia, DR Congo and Senegal. However, we found a different trend for Côte d’Ivoire, Cameroon, Ghana, Sierra Leone and Mali where non-‘slum’ populations seem to have higher prevalence than ‘slum’ populations. Senegal showed a narrower variation in HIV prevalence estimates between the groups.

In Latin America, HIV prevalence among ‘slum’ populations was roughly three times higher than non-‘slum’ populations in Haiti, Dominican Republic and Nicaragua. In India, where we have data from 2015, HIV prevalence disparities between ‘slum’ and non-‘slum’ households were less obvious.

## Discussion

In this retrospective study analysing combined data from five population-level datasets, we found the urban divide between slum and non-slum households is correlated with HIV inequalities, with a significant trend towards the urban slum (ie, poor) suffering higher HIV prevalence rates compared with their urban non-slum (ie, non-poor) counterparts. This phenomenon is likely due to structural drivers of inequalities between urban poor and non-poor populations such as inequalities in wealth, education, employment and housing, which have been well documented to negatively affect HIV outcomes for the poor compared with their richer counterparts, including through higher rates of prevalence and mortality, lower testing uptake and lower levels of HIV knowledge.^27^,^31^ For example, a systematic study on socioeconomic differences and HIV/AIDS mortality in Sub-Saharan Africa demonstrated that persons of low socioeconomic status defined through income level and education had over 50% risk of dying from HIV/AIDS.^28^ Few studies have additionally demonstrated that HIV further exacerbates the effects of poverty.^27 32^ Our study adds to extant literature as one of the first analyses to use multiple publicly available cross-national datasets to assess the combined impact of poverty and urbanity on HIV outcomes. It additionally demonstrates that national estimates of HIV mask nuances in HIV inequalities between the urban ‘slum’ and non-‘slum’ populations.

Beyond disparities within a given urban setting, our cross-regional analyses highlighted similar or worse outcomes in smaller cities (eg, a population between 1 and 5 million) in ESA and WCA than their larger counterparts (eg, a population of >5 million). Possible explanations of this phenomenon could be related to disparities in resources or funding between major and secondary cities; geographical location of Ministries of Health, National AIDS Councils, large academic institutes and research hospitals driving prioritisation of HIV programming; and additional resourcing for capacity building or quicker uptake of innovative programming and interventions in major cities. While the existence of subnational variations in HIV outcomes (ie, mortality, incidence, prevalence) has been well established,^33^,^35^ this is the first global analysis to incorporate urbanity and population size to better understand sub-national variations. Our analysis demonstrates that inequalities between cities, particularly major and secondary cities, may help inform geographically equitable resourcing and financing towards closing 95-95-95 gaps.

This study has some critical limitations. City datasets used were not perfectly comparable as methodologies differed with a mix of surveillance and modelled data. We accounted for this discrepancy by using surveillance data where possible (online supplemental table S3). The datasets reported data for different years spanning from 2019 to 2021, with the majority of the data reported in 2021. The cities included in the study were based on the availability of data, so we were not able to have a representative number of cities from each region for the regional analysis. Additionally, the following countries had a large number of cities in the analysis which might influence results: USA (13), Tanzania (23), Kenya (13), Ghana (24), Ethiopia (28), Democratic Republic of Congo (21), Cameroon (12) and Brazil (13). Assessment of urban ‘slum’ versus non-‘slum’ included merging two separate data sets, DHS and PHIA, both of which use standardised cross-sectional survey methodologies and collect blood samples from subsamples of respondents to test for HIV.^18 19^ In countries where both a DHS and PHIA were conducted within a year of each other, the estimates of HIV prevalence in non-‘slum’ populations were generally consistent, whereas the estimates of HIV in ‘slum’ populations were divergent, with PHIA surveys recording lower HIV prevalence among ‘slum’ households than non-‘slum’ households in several instances (figures2^4^, onlinesupplemental tables S1  S2). While it is possible that the epidemiology of the HIV pandemic in ESA cities changed during the period of measurement, another possible explanation is how the DHS and PHIA approached ‘slum’ communities that were selected to be in the survey, by chance. Depending on the context, extra security and community leader permissions are needed to conduct surveys in slums and informal settlements; in practice, when such a community is selected or if local leaders fail to approve the survey implementation, another cluster within the same city might be randomly or purposefully substituted, potentially leading to systematic bias in the representation of the urban poorest in household surveys. These substitutions are not always documented and reported and are an important topic of study in household survey methodology.^36^

Poverty can be difficult to define in any context, especially urban contexts where asset ownership does not necessarily reflect vulnerabilities to food, housing and other insecurities during economic shocks such as accident, illness, job loss, COVID-19 lockdowns/curfews, or food or fuel price fluctuations. Furthermore, in many countries, poverty from censuses and surveys is known to undercount ‘slum’ dwellers and other vulnerable urban residents.^37^ In our analysis of household survey data, we used ‘slum households,’ a widely accepted metric of poverty which is based on four specific household assets to assess relative and absolute poverty, though this dataset might have under-represented data on the urban poorest as it does not reflect other dimensions of inequality such as those related to income, gender, employment, migration, disability and transportation. Previous studies have found that different forms of wealth, for example, wage economy compared with agricultural economy, are differentially associated with HIV infection.^38^ The present study does not disaggregate wealth along different dimensions, which may conceal varied effects.

Additionally, we would be remiss not to acknowledge that HIV inequalities are most prominent among key populations (ie, men who have sex with men, people who inject drugs, sex workers) and adolescent girls and young women.^16^ Despite key populations making up only 5% of the global population, 70% of new infections in 2021 were among key populations and their sexual partners.^16^ A key limitation in our study was the inability to assess intersecting vulnerabilities between poverty and key and vulnerable populations due to scarcity in data, a particularly important question given that key and vulnerable populations are often economically marginalised and likely disproportionately represented among the urban poorest.^2739^,^41^ Although global and national HIV reporting systems, including several of those included in our study, try to encourage tracking and reporting of HIV indicators among key and vulnerable populations, only a very limited number of cities or national HIV surveys actually collect and report these data in urban areas.

This study presents a novel analysis of HIV inequalities across urban settings of varying sizes and across multiple regions. To our knowledge, this is the largest global analysis of HIV prevalence and incidence data at the city level. By combining and harmonising data from multiple sources, including both surveillance and modelled estimates, the study demonstrates a novel approach for understanding global HIV trends, despite the challenges posed by varying data collection methods and definitions across sources. To our knowledge, this is the first study that examines differences in HIV outcomes by city size, and it reveals significant variations in HIV burden that national averages often conceal. It importantly highlights the heightened HIV prevalence in large secondary cities, where these cities sometimes surpass even capital cities in HIV incidence rates. Additionally, our findings underscore that the urban slum populations bear a disproportionate HIV burden compared with their non-slum counterparts, further emphasising inequities that are largely masked in national surveys.

There is a large scope for additional research to better map out and understand the contexts for geographical and structural HIV inequalities. Further explorations are required to better understand how spatial inequalities affect HIV prevalence rates within countries to guide HIV interventions and policies, particularly as they relate to the secondary cities that experience equal or greater HIV burden compared with major cities, including nuanced regional differences. In addition, it would be informative to understand intersectional vulnerabilities of key and vulnerable populations in the context of poverty, which requires robust collection of subnational subpopulation data. Lastly, understanding the structural drivers underpinning HIV inequalities between urban slum and non-slum populations can inform other health inequalities (eg, pandemics, infectious diseases, non-communicable diseases).

## Conclusion and policy implications

We examined five publicly available datasets and found that the urban divide between the urban slum and non-slum populations is correlated to HIV inequalities. Additionally, our cross-national analyses highlighted similar or worse outcomes in large secondary cities (eg, a population between 1 and 5 million) than their major/capital city counterparts (a population of >5 million or capital cities).

Framed within our global efforts to attain the UNAIDS 95-95-95 targets and the goal to end AIDS by 2030, the intersection of poverty and urbanicity on HIV outcomes reinforces the need for policies to address intersecting social, geographical and structural inequalities such as wealth, education, employment and housing. Additionally, subnational geographic variations in HIV burden can inform strategic human and resource investments to close gaps in the HIV response. Further studies may be undertaken to uncover underlying reasons for variations between capital/major and large secondary cities in countries where this was observed. For example, geographic prioritisation by international donors, variation in availability of services or accessibility of services may play a role in subnational geographic variations between capital/major and large secondary cities. While this study is limited to HIV, similar dynamics between urbanicity, poverty and health inequalities may be relevant to other socially determined diseases such as tuberculosis and hepatitis and warrant further investigation. Ultimately, this study underscores the importance of focused HIV programming accounting for subnational variations and structural drivers of inequalities between urban slum and non-slum populations as a critical measure for closing gaps driving HIV inequalities globally.

## Supplementary material

10.1136/bmjgh-2023-014750 Supplementary file 1

10.1136/bmjgh-2023-014750 Supplementary file 2

10.1136/bmjgh-2023-014750 Supplementary file 3

## Data Availability

At the time of this analysis, Demographic and Health Survey (DHS) and Population-Based HIV Impact Assessment (PHIA) survey data were publicly available to registered users. Other data used for this analysis from Fast Track Cities may be made available upon request.
