# Supplementary material for: Intersectional forces of urban inequality and the global HIV pandemic: a retrospective analysis
Source: BMJ Glob Health. 2025 Apr 9;10(4):e014750. doi: 10.1136/bmjgh-2023-014750 (PMC11987103; doi:10.1136/bmjgh-2023-014750)
Supplement: Supplementary file 1 [file bmjgh-10-4-s001.pdf]

Country	Survey	Year	"Slum" HIV Prevalence		Non-"slum" HIV Prevalence	
**WEST AND CENTRAL AFRICA**						
Burundi	DHS	2017	**3.1**	(2.2 - 4.5)	2.2	(1.2 - 4.2)
Cameroon	DHS	2019	**3.4**	(2.7 - 4.2)	2.5	(1.9 - 3.4)
	PHIA	2018	1.5	(1.2 - 1.8)	**2.3**	(2.0 - 2.7)
Chad	DHS	2015	**4.6**	(3.6 - 5.9)	1.6	(0.6 - 4.0)
Côte d'Ivoire	DHS	2012	**4.7**	(3.8 - 5.8)	4.3	(2.6 - 6.9)
DR Congo	DHS	2014	**1.6**	(1.2 - 2.1)	1.3	(0.5 - 3.4)
Gabon	DHS	2012	**4.7**	(3.9 - 5.7)	3.3	(2.1 - 5.2)
Gambia	DHS	2013	**2.8**	(2.0 - 3.9)	0.6	(0.3 - 1.3)
Ghana	DHS	2014	3.1	(2.3 - 4.1)	**3.9**	(1.8 - 8.1)
Guinea	DHS	2018	**2.2**	(1.6 - 3.1)	1.3	(0.8 - 2.2)
Liberia	DHS	2013	**3.0**	(2.1 - 4.3)	2.6	(1.1 - 6.3)
Mali	DHS	2013	1.8	(1.3 - 2.7)	**2.1**	(1.1 - 3.9)
Senegal	DHS	2017	**0.6**	(0.3 - 1.0)	0.4	(0.2 - 0.8)
Sierra Leone	DHS	2019	2.4	(1.8 - 3.2)	**2.8**	(2.0 - 4.1)
	DHS	2013	2.1	(1.5 - 2.8)	**3.6**	(2.3 - 5.5)
Togo	DHS	2014	**3.7**	(2.9 - 4.7)	3.3	(2.1 - 5.4)
**EASTERN AND SOUTHERN AFRICA**						
Angola	DHS	2016	**2.1**	(1.7 - 2.7)	2.1	(1.1 - 4.0)
Ethiopia	PHIA	2018	**2.2**	(1.9 - 2.6)	0.8	(0.6 - 1.0)
Lesotho	PHIA	2017	10.0	(8.7 - 11.2)	**17.7**	(16.3 - 19.2)
	DHS	2014	**33.8**	(30.0 - 37.9)	22.3	(18.7 - 26.3)
Malawi	DHS	2016	**16.8**	(13.6 - 20.5)	12.1	(9.3 - 15.6)
	PHIA	2016	**8.6**	(7.5 - 9.7)	6.2	(5.2 - 7.1)
Mozambique	DHS	2015	**18.0**	(15.5 - 20.9)	14.4	(11.9 - 17.4)
Namibia	PHIA	2017	**6.4**	(5.2 - 7.6)	4.9	(3.9 - 5.8)
	DHS	2013	**18.1**	(15.9 - 20.6)	6.6	(5.1 - 8.5)
Rwanda	PHIA	2019	2.2	(1.7 - 2.8)	**2.6**	(2.1 - 3.2)
	DHS	2015	**7.4**	(6.2 - 8.8)	4.7	(3.3 - 6.7)
South Africa	DHS	2016	**26.0**	(21.9 - 30.5)	16.4	(13.6 - 19.7)
Tanzania	PHIA	2017	2.7	(2.2 - 3.2)	**3.4**	(3.0 - 3.9)
	DHS	2012	**7.7**	(6.5 - 9.1)	6.0	(4.3 - 8.4)
Uganda	PHIA	2017	**3.8**	(3.1 - 4.5)	3.7	(3.1 - 4.3)
Zambia	DHS	2019	**17.0**	(15.4 - 18.8)	14.0	(12.3 - 15.8)
	PHIA	2016	7.3	(6.4 - 8.2)	**8.4**	(7.5 - 9.3)
Zimbabwe	PHIA	2016	3.8	(3.1 - 4.6)	**11.3**	(10.2 - 12.3)
	DHS	2015	**16.1**	(14.1 - 18.3)	12.7	(10.7 - 14.9)
ASIA						
India	DHS	2016	**0.4**	(0.3 - 0.6)	0.3	(0.2 - 0.4)
**LATIN AMERICA**						
Dominican Republic	DHS	2013	**1.5**	(1.0 - 2.1)	0.5	(0.4 - 0.7)
Haiti	DHS	2017	**2.1**	(1.7 - 2.7)	0.7	(0.3 - 1.5)
	DHS	2012	2.5	(2.0 - 2.9)	**2.8**	(1.8 - 4.4)
Nicaragua	DHS	2012	**0.9**	(0.6 - 1.6)	0.3	(0.1 - 0.8)
