# Supplementary material for: Intersectional forces of urban inequality and the global HIV pandemic: a retrospective analysis
Source: BMJ Glob Health. 2025 Apr 9;10(4):e014750. doi: 10.1136/bmjgh-2023-014750 (PMC11987103; doi:10.1136/bmjgh-2023-014750)
Supplement: Supplementary file 2 [file bmjgh-10-4-s002.pdf]

source	region	iso	country	year	residence	n_hiv_unwgt	n_all_unwgt	hiv_prev	hiv_prev_l	hiv_prev_u	StdErr
DHS	ESA	24	Angola	2016	nonslum	31	1306	2.06	1.05	3.99	0.699
DHS	ESA	24	Angola	2016	slum	158	5874	2.14	1.71	2.68	0.245
DHS	WCA	108	Burundi	2017	nonslum	32	1355	2.20	1.15	4.16	0.720
DHS	WCA	108	Burundi	2017	slum	61	2125	3.15	2.17	4.55	0.593
PHIA	WCA	120	Cameroon	2018	nonslum	270	6289	2.34	1.95	2.72	0.196
PHIA	WCA	120	Cameroon	2018	slum	183	4855	1.50	1.21	1.78	0.145
DHS	WCA	120	Cameroon	2019	nonslum	81	3099	2.53	1.89	3.37	0.371
DHS	WCA	120	Cameroon	2019	slum	146	4220	3.37	2.69	4.22	0.387
DHS	WCA	148	Chad	2015	nonslum	8	302	1.56	0.60	4.01	0.759
DHS	WCA	148	Chad	2015	slum	94	2399	4.60	3.58	5.89	0.583
PHIA	WCA	384	Côte d'Ivoire*	2018	nonslum	1	14	0.00	0.00	0.01	0.004
PHIA	WCA	384	Côte d'Ivoire*	2018	slum	268	9080	2.94	2.46	3.42	0.242
DHS	WCA	384	Côte d'Ivoire	2012	nonslum	50	1078	4.27	2.60	6.92	1.061
DHS	WCA	384	Côte d'Ivoire	2012	slum	132	2841	4.71	3.81	5.82	0.507
DHS	LA	214	Dominican Republic	2013	slum	66	3763	1.46	1.03	2.07	0.258
DHS	LA	214	Dominican Republic	2013	nonslum	67	9108	0.54	0.39	0.75	0.090
DHS	WCA	180	DR Congo	2014	nonslum	7	580	1.35	0.53	3.38	0.637
DHS	WCA	180	DR Congo	2014	slum	87	5534	1.58	1.21	2.06	0.214
PHIA	ESA	748	Eswatini*	2017	nonslum	6	30	0.29	0.04	0.54	0.128
PHIA	ESA	748	Eswatini*	2017	slum	719	2265	30.87	27.16	34.58	1.884
PHIA	ESA	231	Ethiopia	2018	nonslum	183	5942	0.82	0.64	1.01	0.094
PHIA	ESA	231	Ethiopia	2018	slum	431	13194	2.23	1.86	2.60	0.187
DHS	WCA	266	Gabon	2012	nonslum	69	2011	3.33	2.13	5.16	0.749
DHS	WCA	266	Gabon	2012	slum	254	5205	4.75	3.92	5.75	0.462
DHS	WCA	270	Gambia	2013	nonslum	11	1318	0.60	0.28	1.28	0.232
DHS	WCA	270	Gambia	2013	slum	49	2089	2.79	1.97	3.95	0.492
DHS	WCA	288	Ghana	2014	nonslum	7	200	3.87	1.81	8.06	1.473
DHS	WCA	288	Ghana	2014	slum	62	2083	3.08	2.31	4.09	0.449
DHS	WCA	324	Guinea	2018	nonslum	16	1131	1.30	0.76	2.20	0.351
DHS	WCA	324	Guinea	2018	slum	50	2227	2.23	1.58	3.14	0.390
DHS	LA	332	Haiti	2012	nonslum	24	972	2.84	1.83	4.36	0.625
DHS	LA	332	Haiti	2012	slum	182	6696	2.46	2.05	2.94	0.228
DHS	LA	332	Haiti	2017	nonslum	6	667	0.65	0.29	1.49	0.274
DHS	LA	332	Haiti	2017	slum	143	6328	2.12	1.66	2.69	0.258

DHS	AP	356	India	2016	nonslum	85	31297	0.31	0.22	0.44	0.055
DHS	AP	356	India	2016	slum	137	37563	0.43	0.31	0.58	0.067
PHIA	ESA	426	Lesotho	2017	nonslum	939	3514	17.72	16.29	19.15	0.729
DHS	ESA	426	Lesotho	2014	nonslum	146	641	22.29	18.72	26.33	1.937
DHS	ESA	426	Lesotho	2014	slum	395	1287	33.85	29.97	37.95	2.032
PHIA	ESA	426	Lesotho	2017	slum	538	1793	9.97	8.74	11.21	0.630
DHS	WCA	430	Liberia	2013	nonslum	9	415	2.63	1.07	6.29	1.185
DHS	WCA	430	Liberia	2013	slum	69	2702	3.03	2.12	4.31	0.545
PHIA	ESA	454	Malawi	2016	nonslum	493	2908	6.19	5.25	7.13	0.480
DHS	ESA	454	Malawi	2016	nonslum	159	1324	12.10	9.29	15.62	1.604
DHS	ESA	454	Malawi	2016	slum	262	1842	16.79	13.65	20.48	1.737
PHIA	ESA	454	Malawi	2016	slum	568	3608	8.57	7.48	9.66	0.553
DHS	WCA	466	Mali	2013	nonslum	13	670	2.05	1.08	3.87	0.667
DHS	WCA	466	Mali	2013	slum	33	1854	1.84	1.26	2.69	0.355
DHS	ESA	508	Mozambique	2015	nonslum	219	1451	14.40	11.87	17.36	1.392
DHS	ESA	508	Mozambique	2015	slum	673	3638	18.04	15.49	20.91	1.377
PHIA	ESA	516	Namibia	2017	nonslum	340	3497	4.86	3.89	5.83	0.494
PHIA	ESA	516	Namibia	2017	slum	545	3459	6.41	5.25	7.58	0.592
DHS	ESA	516	Namibia	2013	nonslum	129	1739	6.58	5.08	8.47	0.857
DHS	ESA	516	Namibia	2013	slum	439	2521	18.12	15.91	20.56	1.186
DHS	LA	558	Nicaragua	2012	nonslum	3	582	0.25	0.08	0.79	0.147
DHS	LA	558	Nicaragua	2012	slum	20	1989	0.94	0.56	1.57	0.246
PHIA	ESA	646	Rwanda	2019	nonslum	208	5175	2.62	2.07	3.16	0.277
PHIA	ESA	646	Rwanda	2019	slum	154	2456	2.24	1.69	2.79	0.280
DHS	ESA	646	Rwanda	2015	nonslum	49	1140	4.71	3.29	6.70	0.853
DHS	ESA	646	Rwanda	2015	slum	145	2124	7.36	6.16	8.78	0.662
DHS	WCA	686	Senegal	2017	nonslum	12	2759	0.38	0.18	0.81	0.146
DHS	WCA	686	Senegal	2017	slum	27	3629	0.57	0.33	0.99	0.161
DHS	WCA	694	Sierra Leone	2013	nonslum	28	893	3.62	2.35	5.54	0.790
DHS	WCA	694	Sierra Leone	2013	slum	105	5063	2.06	1.49	2.83	0.335
DHS	WCA	694	Sierra Leone	2019	nonslum	33	1058	2.85	1.99	4.05	0.515
DHS	WCA	694	Sierra Leone	2019	slum	99	4237	2.40	1.80	3.21	0.355
DHS	ESA	710	South Africa	2016	slum	291	1097	25.96	21.92	30.46	2.177
DHS	ESA	710	South Africa	2016	nonslum	251	1390	16.42	13.62	19.67	1.539
PHIA	ESA	834	Tanzania	2017	nonslum	395	5988	3.41	2.96	3.85	0.227
PHIA	ESA	834	Tanzania	2017	slum	352	4406	2.68	2.18	3.18	0.254
DHS	ESA	834	Tanzania	2012	nonslum	55	1213	6.04	4.30	8.44	1.039
DHS	ESA	834	Tanzania	2012	slum	226	2855	7.67	6.46	9.08	0.664
DHS	WCA	768	Togo	2014	nonslum	24	716	3.35	2.06	5.39	0.820
DHS	WCA	768	Togo	2014	slum	98	2606	3.69	2.90	4.70	0.453

PHIA	ESA	800	Uganda	2017	nonslum	293	3822	3.67	3.09	4.26	0.299
PHIA	ESA	800	Uganda	2017	slum	325	4193	3.84	3.13	4.55	0.360
PHIA	ESA	894	Zambia	2016	nonslum	757	4252	8.43	7.53	9.34	0.460
DHS	ESA	894	Zambia	2019	nonslum	418	2897	13.97	12.29	15.84	0.905
DHS	ESA	894	Zambia	2019	slum	1083	6526	17.03	15.40	18.80	0.865
PHIA	ESA	894	Zambia	2016	slum	657	4277	7.27	6.35	8.20	0.469
PHIA	ESA	716	Zimbabwe	2016	nonslum	821	4976	11.26	10.24	12.28	0.520
PHIA	ESA	716	Zimbabwe	2016	slum	261	1433	3.85	3.06	4.64	0.403
DHS	ESA	716	Zimbabwe	2015	nonslum	396	2910	12.66	10.74	14.86	1.046
DHS	ESA	716	Zimbabwe	2015	slum	675	3966	16.06	14.05	18.29	1.077
*Excluded due to n_unwgt <50											
