# Supplementary material for: Intersectional forces of urban inequality and the global HIV pandemic: a retrospective analysis
Source: BMJ Glob Health. 2025 Apr 9;10(4):e014750. doi: 10.1136/bmjgh-2023-014750 (PMC11987103; doi:10.1136/bmjgh-2023-014750)
Supplement: Supplementary file 3 [file bmjgh-10-4-s003.pdf]

City	Country	Prevalence		Incidence	
		FTC	Naomi	FTC	NAOMI
Kinshasa	DR Congo	--	0.6	0.48	17
Accra	Ghana	1.7	1.4	1.1	61
Nairobi	Kenya	6	3.4	--	106
Blantyre	Malawi	17.37	10.6	0.42	221
Lagos	Nigeria	--	0.7	0.49	20
Lusaka	Zambia	--	10.9	1783	322
